# Transgressive phenotypes from outbreeding between the *Trichoderma reesei* hyper producer RutC30 and a natural isolate

**DOI:** 10.1128/spectrum.00441-24

**Published:** 2024-08-20

**Authors:** Laetitia Chan Ho Tong, Etienne Jourdier, Delphine Naquin, Fadhel Ben Chaabane, Thiziri Aouam, Gwladys Chartier, Itzel Castro González, Antoine Margeot, Frederique Bidard

**Affiliations:** 1 Biotechnology Department, IFP Energies nouvelles (IFPEN), 92852 Rueil-Malmaison, France; 2 Institute for Integrative Biology of the Cell (I2BC), Université Paris-Saclay, CEA, CNRS, 91198 Gif-sur-Yvette, France; University of Natural Resources and Life Sciences, Vienna, Austria

**Keywords:** *Trichoderma reesei*, sexual reproduction, cellulases, transgressive phenotype, outbreeding

## Abstract

*Trichoderma reesei,* the main filamentous fungus used for industrial cellulase production, was long considered to be asexual. The recent discovery of the mating type locus in the natural isolate QM6a and the possibility to cross this sterile female strain with a fertile natural female strain opened up a new avenue for strain optimization. We crossed the hyperproducer RutC30 with a compatible female ascospore-derived isolate of the wild-type strain CBS999.97 and analyzed about 300 offspring. A continuous distribution of secreted protein levels was observed in the progeny, confirming the involvement of several mutated loci in the hyperproductive phenotype. A bias toward *MAT1-2* strains was identified for higher producers, but not directly linked to the Mating-type locus itself. Transgressive phenotypes were observed in terms of both productivity and secretome quality, with offspring that outperform their parents for three enzymatic activities. Genomic sequences of the 10 best producers highlighted the genetic diversity generated and the involvement of parental alleles in hyperproduction and fertility.

**IMPORTANCE:**

The filamentous fungus *Trichoderma reesei* produces cellulolytic enzymes that are essential for the hydrolysis of lignocellulosic biomass into monomerics sugars. The filamentous fungus *T. reesei* produces cellulolytic enzymes that are essential for the hydrolysis of lignocellulosic biomass into monomerics sugars, which can in turn be fermented to produce second-generation biofuels and bioproducts. Production performance improvement, which is essential to reduce production cost, relies on classical mutagenesis and genetic engineering techniques. Although sexual reproduction is a powerful tool for improving domesticated species, it is often difficult to apply to industrial fungi since most of them are considered asexual. In this study, we demonstrated that outbreeding is an efficient strategy to optimize *T. reesei*. Crossing between a natural isolate and a mutagenized strain generated a biodiverse progeny with some offspring displaying transgressive phenotype for cellulase activities.

## INTRODUCTION

For centuries, properties of domesticated plants and animals have been improved through crossbreeding to produce offspring with advanced characteristics. By contrast, sexual reproduction has been little used for the improvement of industrial fungal strains, since most of them were categorized in the 20% fungi with no known sexual state (1). Reports of sexual reproduction in biotechnologically relevant fungi (2–6) open new possibilities in strain development: combination of beneficial genes in offspring, removal of deleterious mutations accumulated through asexual propagation, and identification of genes of interest by classical genetics combined with genomics (BSA-seq).

Derivatives of the natural isolate QM6a of the filamentous fungus *Trichoderma reesei*, which was isolated in the Solomon Islands during World War II (7), are among the most efficient producers of cellulases used in industry (8). Long considered asexual (9), improving its enzyme production has relied mainly on classical random mutagenesis (10–13) and, more recently, genetic engineering techniques (14–16). These techniques have proved useful, but each has its limitations: random mutagenesis introduces unwanted mutations whose accumulation can lead to phenotypic instability, and recombinant DNA produces genetically modified organisms (GMO) that trigger environmental constraints for industrial facilities in some parts of the world. The discovery of a heterothallic sexual cycle in a sexual counterpart isolate of *T. reesei* called CBS999.97 (17) highlighted another avenue for strain improvement (6). Successful sexual reproduction in heterothallic species requires compatible partners carrying a different mating type locus, *MAT1-1* or *MAT1-2*. The *T. reesei* QM6a strain, which gives rise to all derived strains used in industry today, carries the *MAT1-2* idiomorph and is unfortunately female sterile (6). However, QM6a or its derivatives, acting as a male mating partner, can be crossed with a *MAT1-1* strain derived from a single ascospore of the strain CBS999.97 (6).

Mobilizing natural biodiversity is a powerful strategy for improving industrial strains. A new natural isolate may possess certain industrially relevant traits (18), and sexual reproduction could transfer these traits to industrial strains, creating new individuals with novel properties. Sexual reproduction, as a tool for strain improvement, has been widely applied in *Saccharomyces cerevisiae* (19, 20). In *P. chrysogenum*, crossing the high penicillin and chrysogenin producer Q176 (2) with a wild-type strain lacking detectable chrysogenin results in offspring that combine a high penicillin titer without producing yellow pigment. In addition, genome-wide recombination that occurs during the sexual cycle between mating partners can generate substantial diversity in the offspring and produce transgressive phenotypes that exceed the parental ones (21). Transgressive segregation, namely the generation of extreme phenotypes compared with parents, has been described mainly in plants and animals (22, 23) but has also been reported in fungi (24–26).

The *T. reesei* RutC30 strain (13) is the result of three rounds of random mutagenesis of the QM6a and is one of the best cellulase producers in the public domain (11). Genetic experiments and whole genome analysis of this strain have revealed numerous mutations including translocations, large deletions, and more than 200 single nucleotide variants (SNVs) (27–30). Only a few of these mutations have been linked to hyperproduction phenotype: the truncation of the catabolite repressor Cre1 (31), a missense mutation leading to the truncation of the transcription factor ACE3 (32), a frameshift mutation in the glucosidase II alpha subunit gene *gls2* (33), and an SNV in the transcription factor BglR (34). Other mutations may have a significant yet unknown effect on cellulase production (28), but it is unlikely that all the mutations identified in RutC30 are involved in cellulase production. Furthermore, some of them may have a deleterious impact on production, growth, and genetic stability.

In this study, we undertook the breeding of a compatible and female fertile wild-type strain derived from CBS999.97 and the hyperproducer strain RutC30. This cross, between two genetically divergent strains, was expected to induce genomic recombination and produce offspring with high phenotypic variation (35). The descendants of this cross were characterized by their protein production capacity. A higher productivity bias by *MAT1-2* strains observed in the progeny does not appear to be directly related to the transcription factors of the mating type locus. Further in-depth phenotypic analysis of 10 of the top producers revealed transgressive phenotypes with higher cellulase, β-glucosidase, and cellobiohydrolase activities in some of the progeny compared with the parental strains, demonstrating the effectiveness of the outbreeding approach. The sequencing of the genomes of these 10 strains confirmed the relevance of the *ace3*, *cre1,* and *bglR* mutations in the hyperproducer phenotype of the RutC30 strain.

## MATERIALS AND METHODS

### Strains and growth conditions

The *T. reesei* strains used in this study with their reference and their mating type are listed in Table 1. Strain propagation and purification were performed on Potato Dextrose Agar (PDA). For submerged cultures, strains were grown in potato dextrose broth. All strains are maintained as a conidial suspension in CTS50 (0.4M saccharose; 0.1M Tris·HCl; 50 mM CaCl_2_; pH 7.5) and frozen at −80°C.

**TABLE 1:** List of the *T. reesei* strains used in this study

Strain name	Mating type	Sources
RutC30	*Mat1-2*	(ATCC 56765)
CBS999.97	*Mat1−1/Mat1-2*	(ATCC 204423)
QM6a Mat1-1	*Mat1-1*	(36)
GJS 85–249	*Mat1-1*	(36)
QM6a	*Mat1-2*	(ATCC 13631)
A2	*Mat1-1*	This study
RutC30 Mat1-1	*Mat1-1*	This study
B31	*Mat1-2*	This study
RuA10	*Mat1-2*	This study
RuA70	*Mat1-2*	This study
RuA74	*Mat1-2*	This study
RuA82	*Mat1-2*	This study
RuA97	*Mat1-2*	This study
RuA128	*Mat1-2*	This study
RuA141	*Mat1-2*	This study
RuA148	*Mat1-2*	This study
RuA149	*Mat1-1*	This study
RuA156	*Mat1-1*	This study

### DNA manipulation

Sequences of oligonucleotides used in this study are listed in Table S1 (supplementary data). The genomic DNA for the sequencing of the wild-type strain A2 was extracted according to (37) . Other genomic DNAs were extracted using the Nucleospin Soil Genomic DNA kit (Macherey Nagel). Mating types were identified by amplifying an internal part of the locus with oligonucleotides Mat1-1-F-intern/Mat1-1-R-intern for *MAT1-1* and Mat1-2-F-intern/Mat1-2-R-intern for *MAT1-2*. To construct the *MAT1-1* replacement cassette, the locus *MAT1-1* was amplified from the QM6a *MAT1-1* IDC1 strain provided by Dr. B. Seiboth (Vienna University of Technology, Austria). This strain was previously constructed by (36) by amplifying the *MAT1-1* locus from strain G.J.S. 85–249 and introducing the hygromycin resistance gene (*hph*) as a selection marker. The *MAT 1–1* locus was amplified in two fragments (F1 and F2) of sizes 5 and 6.1 kb with, respectively, the oligonucleotides PR73/PR74 and PR75/PR76 and assembled by recombinational cloning of yeast (38). Approximately 19 bp overlapping the yeast shuttle vector pRS426 (URA+) or F1/F2 fragments were introduced by PCR at each flanking end to allow this homologous recombination. The yeast transformation procedure was performed as described by (39). Plasmid pRS426-MAT1-1 recovered from yeast transformation was introduced and amplified in chemically competent NEB 10-beta *E. coli* cells (New England Biolabs). The integrity of the *MAT1-1* cassette was checked by sequencing (Eurofins MGW). For *T. reesei* transformation, the *MAT1-1* cassette was purified after digestion of pRS426-MAT1-1 by the restriction enzymes *SacI* and *PfoI*. Five micrograms of the cassette were used for the protoplast transformation of *T. reesei*.

### *T. reesei* transformation

*T. reesei* RutC30 was the target for the replacement of *MAT1-2* with the *MAT1-1* locus. Preparation and transformation of protoplasts are performed as previously described (40). Homokaryotic transformants are obtained by plating conidia on a selective medium. The correct integration of the *MAT1-1* cassette replacing *MAT1-2* was first verified by sexual crosses with the female fertile strains B31 and A2. Only strains with a *MAT1-1* locus capable of generating progeny with B31 and not with A2 were selected. Insertion at the correct locus was verified with primer pairs P129/P130 and P133/P134 amplifying the 5′ or 3′ flanking regions and the *MAT1-1* cassette. Two independent clones were isolated and analyzed as biological replicates.

### Sexual crosses

The sexual crossing by confrontation assay was performed on PDA with incubation at 24°C and an alternate of 12 h light and 12 h dark. The confrontation was also used to phenotypically determine the mating type of strain (mating-type test): the strain to be tested was placed in the middle of a Petri dish and the *MAT1-1* strain A2, and the *MAT1-2* strain B31 was inoculated on both opposite edges of the plate. Stromata only appeared at the confrontation between the tested strain and its compatible partner.

To recover ascospores shot to the plate lid without contamination by airborne conidia, we took advantage of the cell wall differences that exist between sexual and asexual spores. In *T. reesei*, the ascospores are hyaline (41), whereas the conidia are pigmented green. Pigmented spores are generally thick-walled and require specific triggers to initiate germination, whereas unpigmented spores are generally thin-walled and capable of immediate germination (42). Thus, when conidia and ascospores were put to germinate and grow on water agar at 30°C, after 48 h of incubation, only a few conidia germinated without further development. In contrast, ascospores germination leads to a mycelium capable of conidia production.

For single ascospore isolation, ascospores were harvested by washing the lid with 2 mL of CTS50, counted, and diluted in order to spread approximately 30 ascospores per Agar + H_2_O Petri dish (20 g/L). After incubating the plates at 30°C for at least 48 h, mycelia from a single ascospore were picked on PDA and purified. The isolated progeny from ascospores were stored at −80°C at 10^6^ conidia/mL in CTS50.

### Screening process

For primary screening, progenies were cultivated in 24 well plates containing 2 mL of F45 medium (43) containing 10 g/L of lactose and 10 g/L of microcrystalline cellulose (TechnoCel C10, CFF, Belgique). Each well was inoculated with conidia from the respective progeny and cultivated for a week at 30°C with an agitation of 125 rpm. Each culture supernatant was collected, centrifuged for 10 min at 13,000 rpm to sediment the cells and debris, and frozen at −20°C until the protein concentration was determined by the Bradford method (Quick Start Bradford Protein Assay, Bio-Rad) with BSA as a standard.

Secondary screening was performed using the fed-flask culture protocol. This protocol, as well as Filter Paper activity, protein concentration, cellobiohydrolase, and β-glucosidase activities, was performed according to (44).

### Genome sequencing and analysis

For the A2 strain, paired-end and mate-pair libraries were constructed using, respectively, the Illumina “Nextera DNA Library Prep Kit” and the “Nextera Mate Pair Library preparation kit,” by the “Gel-plus 3–5 kb” protocol, according to the manufacturer’s recommendations. Library quality was assessed on a Bioanalyzer (Agilent Technologies) prior to sequencing on an Illumina Miseq instrument. 8.3 M and 9.7 M paired-end reads of 250 nt were produced, respectively, from paired-end and mate-pair libraries. Image analysis, base calling, and quality check were performed with the Illumina data analysis pipeline. Read quality was checked by FastQC 0.10.1. Adaptor sequences were trimmed using Cutadapt-1.3, and the internal adaptors of the mate-pair fragments were trimmed using Nextclip-1.3.1. A homemade filtering script has been added to keep the longest 3’ part of each read having a base quality greater than 30 for all nucleotides. Prior to read assembly, the fastx_reverse_complement command in fastx-toolkit was performed to reverse-complement read1 and read2 from mate-pair fragments. A Velvet_1.2.10 assembly (45) from both paired-end and mate-pair reads by using a k-mer length of 89 and the scaffolding option produced 54 contigs/scaffolds whose analysis reveals a N50, which equals to 1.4 Mb and the longest contig equals to 2.7 Mb. Data are available at NCBI Sequence Read Archive under the accession number PRJNA1031156.

For progeny strains, library preparation and Illumina sequencing with 2 × 150 bp paired-end read mode were achieved by Eurofins Genomics Europe Sequencing GmbH (Konstanz, Germany). Read quality was checked by FastQC on Galaxy platform (https://usegalaxy.org/). All the following steps have been carried out on Geneious Prime®. Paired sequences were trimmed and quality-filtered using BBDuk Trimmer (version 1.0, Biomatters Ltd.). Alignment using Geneious assembler was performed simultaneously on both parental genomes to identify chromosomal fragments belonging to one or another parent. Data are available at NCBI Sequence Read Archive under the accession number PRJNA1031805.

## RESULTS AND DISCUSSION

### Isolation of a wild-type strain able to cross with the QM6a and RutC30 strains

Of all the *Hypocrea jecorina* strains described in the literature (6, 17), only the ascospores of *Hypocrea jecorina* CBS999.97, a vegetatively compatible mixture of both mating types capable of forming mature stromata, appear to be able to produce fertile crosses with other wild-type strains (46). Since the isolate QM6a and its derivatives are *MAT1-2* (6), it was therefore necessary to isolate a *MAT1-1* strain from the progeny of the heterokaryotic strain CBS999.97. Single ascospore cultures obtained from CBS999.97 displayed macroscopic phenotypic differences, indicating a genomic polymorphism in the nuclei of the heterokaryon (supplementary data, Fig. S1). Consistent with (6), a 1:1 segregation was observed for mating type.

Individual isolates were tested for mating type and female fertility by mating confrontation experiments with the female sterile strain *MAT1-2* QM6a. Isolates with phenotypes closest to QM6a, i.e., flat mycelium with dark green conidia, were selected, and a representative isolate of a *MAT1-1* strain was chosen and called A2. A compatible partner called B31, also originating from *T. reesei* CBS999.97, was selected by confrontation with A2 strain. Their mating types were confirmed by a PCR amplification of an internal fragment of the expected region and the absence of amplification of the opposite one (supplementary data, Fig. S2). Finally, the sexual compatibility of the A2 and RutC30 strains was successfully verified.

Sequence heterozygosity in the progeny of the heterokaryotic strain CBS999.97 has been suspected to be responsible for the production of viable and non-viable segmentally aneuploid ascospores (46). One of the observed karyotype structures is identical to that of QM6a while the other displays a novel chromosomal arrangement (called *re*), consisting of exchanges in telomeric parts of chromosomes II and IV (30). The issue of non-viable ascospores could be accentuated with strains improved by mutagenesis as their genomes have acquired multiple chromosomal rearrangements (21). In light of these data, the karyotype of the A2 strain was examined by a sequencing experiment combined with a *de novo* assembly to ensure that it was a QM6a type. A matrix similarity plot (supplementary data, Fig. S3) of A2 contigs against QM6a chromosomes (30, 47) displayed a collinearity along the whole genome. This result points to a similarity in chromosomal structure between the two strains. Furthermore, an analysis of contigs 117 and 129 (NCBI assembly database ASM200658v1), which covers the recombinant regions, does not indicate any gaps or translocations. Thus, we can conclude that the A2 strain has a QM6a-type karyotype.

### Progeny screening displayed a continuous distribution of protein production and a bias for higher production in the *MAT1-2* strains

**Fig 1:**
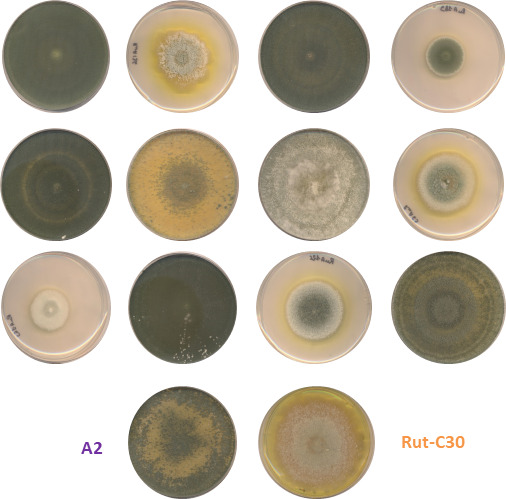
Phenotypes of offspring obtained from crosses of A2 with RutC30. Ascospore-derived isolates and parents were cultivated on PDA, 7 days at 30°C with an alternation of 6 h of darkness for 18 h of light.

Crossing experiments between the RutC30 and the A2 strains were carried out, and ascospores were collected, set to germinate immediately or frozen for later use. To avoid sampling bias due to growth differences between colonies, ascospores were plated on water agar, and all those germinating on a Petri dish were picked up. Two crosses followed by single ascospore isolation campaigns yielded 295 purified descendants. The diversity of macroscopic phenotypes observed on the plate illustrates the evidence of recombination in the progeny (Fig. 1). These strains were then screened for enzyme secretion.

**Fig 2:**
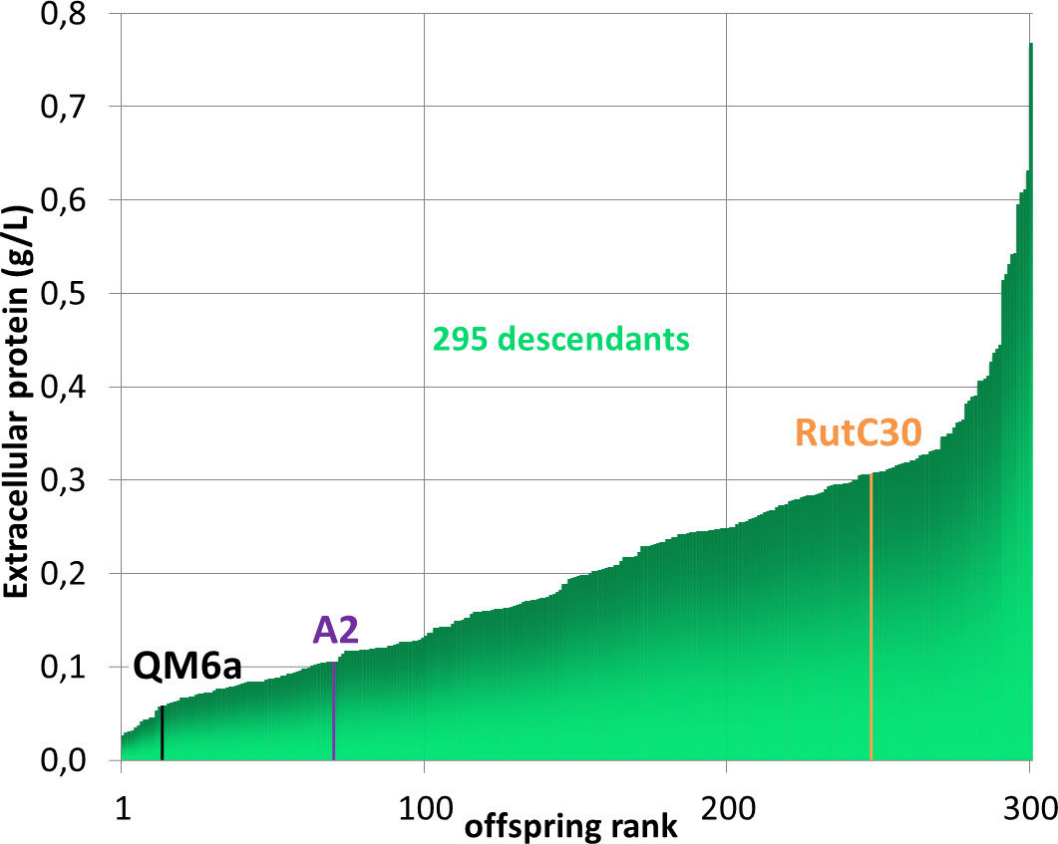
Distribution of extracellular protein concentration of the 295 progeny from A2 X RutC30 crosses. Protein concentrations in the supernatants were quantified using Bradford protein assay. Colored lines indicate control strains.

The cultures were grown in 24-well plates in a medium containing a mixture of cellulose and lactose as carbon and inducer sources. To minimize differences in biomass among strains, the amount of protein secreted in the supernatant was quantified after 7 days of culture (Fig. 2). This screening method for protein production does not consider differences in biomass growth. Therefore, it cannot be excluded that some of the low producers have a slow growth rate. Despite these pitfalls, this method can be used to screen a large number of strains. As expected, the RutC30 strain produced more extracellular proteins than the wild-type strains. A2 has a higher production than QM6a, which confirms that the latter is not the best natural isolate for cellulase producers, as already shown by (48). More surprisingly, some progeny (17%) exhibited higher protein production than the industrial reference RutC30 (up to two times more), whereas others produced less than wild-type A2 (23%). Most of the offspring displayed an intermediate protein concentration between both parental strains (60%). The concentrations of the secreted proteins have a continuous distribution, consistent with the involvement of several genes in the hyperproducer phenotype of RutC30.

Mating-type loci could be involved in many biological processes such as metabolism, morphology, or secondary metabolism in various fungi (49–53). In *T. reesei*, the mating-type transcription factor *MAT1-2-1* has been shown to interact directly with the key transcriptional activator of cellulase XYR1, and deletion of *MAT1-2-1* results in reduced cellulase expression (54). Controversially, gene expression studies of mating type-dependent regulation in QM6a, CBS999.97 *MAT1-1,* and *MAT1-2* and two backcrossed derivatives of the QM6a background (35) revealed that the targets of the mating-type transcription factors are themselves and the pheromone and receptor genes (55).

To investigate the relationship between mating type and cellulase production in the progeny, the mating type of each offspring was determined by a confrontation mating-type test using A2 and B31 as strain testers. Surprisingly, 73% of the offspring were *MAT1-1* and 27% *MAT1-2,* whereas 50% of each mating type was expected as in the progeny of the CBS999.97 strain (6).

The offspring were divided into three equal subpopulations (98, 98, and 99 strains, respectively) based on protein production (Table 2), and the percentage of each mating type in the three groups was calculated. We found that the proportion of *MAT1-1* individuals was highest in the low-producer class, whereas the proportion of *MAT1-2* individuals was highest in the high-producer class. As the concentration of secreted proteins increases, the number of *MAT1-2* individuals per class increases (Table 2). The statistical test of χ2 confirms the existence of a bias toward a mating type depending on the level of cellulase secretion (χ2 = 11.5, df = 2, *P* < 0.005).

**TABLE 2:** Mating-types distribution by protein production groups^*a*^

Mating type	Low	Medium	High	All
***MAT1-1***	83 (84,3%)	69 (70,4%)	63 (63,6%)	**215** (**73%**)
***MAT1-2***	15 (15,3%)	29 (29,6%)	36 (36,4%)	**80** (**27%**)
**All**	**98** (**33,2%**)	**98** (**33,2%**)	**99** (**33,6%**)	**295** (**100%**)

^*a*^ The offspring were divided into three groups of equal size according to their extracellular protein production. In each group, the ratio of each mating type was determined.

### No differences in protein production between RutC30 *MAT1-1* and *MAT1-2* strains in the fed-flask experiment

**Fig 3:**
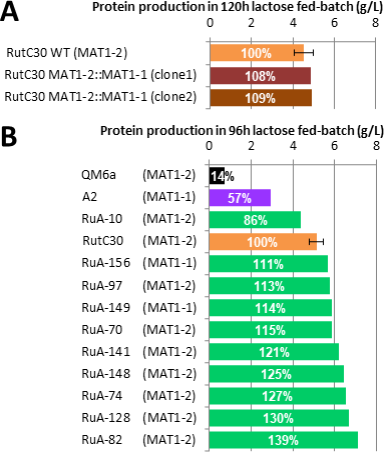
Protein production in lactose fed-batch. A: Production of two independent transformants of RutC30 *MAT1-1* compared with the original RutC30 *MAT1-2*. B: Production of the 10 selected strains from the first screening, the wild-type QM6a, and A2 strains compared with the original RutC30 *MAT1-2*.

To further understand the involvement of mating type in the production of cellulase, a RutC30 *MAT1-1* strain was constructed by replacing the *MAT1-2* locus with the *MAT1-1* locus of the QM6a *MAT1-1* strain (36), which contains the *MAT1-1* locus from the ATCC 13631 strain and the *hph* selection cassette. The integration of the *MAT1-1* cassette was verified by PCR analysis, and a crossing experiment confirmed its ability to mate with the fertile *MAT1-2* B31 and its inability to mate with A2. The protein production of the original RutC30 and two independent RutC30 *MAT1-1* strains was investigated using a miniaturized fed-batch protocol developed by (44). This method consists of two steps: first, biomass production on glucose, followed by cellulase production with a lactose fed-batch combining induction, carbon limitation, and pH stabilization. In this protocol, strains can be compared on the basis of final protein concentration because the rate of protein production is linear throughout the feeding phase (43). A slight but non-significant difference in extracellular protein production was observed between the three strains (Fig. 3A), meaning that the previously observed bias linked to the mating type locus is no longer visible under this hyperproducing condition.

Another potential source for this bias may be the lack of recombination around the mating-type locus. This phenomenon is widespread in fungi, although the mechanism of suppression and the size of the region vary between species (56). The source of the observed bias could then be due to beneficial mutations that previously appeared in RutC30 segregating with *MAT1-2*. In crossbreeding experiments carried out to identify the locus responsible for female sterility in QM6 (36), the authors noticed an enrichment of sequence differences uncorrelated with fertility and located on scaffolds belonging to the mating-type chromosome. The authors suggested an absence of meiotic recombination in the region surrounding the mating type locus as already described i*n Podospora anserina* (56, 57). In this filamentous fungus, the NRR comprises 229 genes and 687 polymorphisms while 10% of the genes show a different transcriptomic profile between the two mating-type strains. In *T. reesei*, no survey has been conducted to determine the size of the inhibition zone, but crosses experiments carried out to identify the locus responsible for sterility have helped define a potential NRR (36). Taking advantage of the telomere-to-telomere assembly of the QM6a genome (47), we located the polymorphisms identified by (36) and defined a 1.5 Mb region with potential meiotic recombination suppression (from ID55213/TrC1091C to ID46816/TrC1576C) including the mating-type locus and called the non-recombination region (NRR). Among the 454 genes of the NRR, seven genes had SNV-type mutations: four in exons, one in intron, one in promoter, and one in terminator (Supplementary Data, Table S2). None of these genes can be directly linked to an increase in protein production, although a possible role in the hyperproductivity of the strains cannot be completely ruled out. The allelic version of the genes located in this region may also contribute to the observed production differences. As it has been shown in *P. anserina* that the NRR can vary between species and strains (56), it would be relevant to define the non-recombination zone of the cross performed in our study in order to identify precisely the genes involved. Indeed, genes important for cellulase production present in the vicinity of the predicted NRR could be part of the actual NRR.

In addition, a transcriptomic study of the RutC30 *MAT 1–1* and RutC30 *MAT1-2* strains would also be useful to explore a whole-genome transcriptional profile under cellulase production conditions.

### Characterization in industrial-like conditions of 10 strains selected among the best producers

To refine the first screening results, 10 strains among the best producers were selected and cultured with the fed-flask protocol described above, with QM6a, A2, and RutC30 as controls. This set includes two *MAT1-1* (RuA-156 and RuA-149) and eight *MAT1-2* (RuA-10, RuA-70, RuA-74, RuA-82, RuA-97, RuA-128, RuA-141, and RuA-148) strains. The production rate was normalized using the hyperproducing strain RutC30 as a reference (Fig. 3B).

The ability of A2 to produce more proteins than QM6a is confirmed with a four times higher rate (57% compared with 14%). Compared with the hyper-producer RutC30, the production rate of A2 is only half as high, whereas QM6a is six times lower. Apart from RuA-10, the selected offspring have a similar or higher production than the reference strain RutC30 (111% to 139%). The two *MAT1-1* strains displayed only a slight improvement in protein production, whereas the best strains are *MAT1-2* type.

To assess the quality of the enzyme cocktail, the main enzymatic activities were measured in the supernatant: cellulase activity by filter paper assay (FP), cellobiohydrolase activity due to Cel7A/CBHI, and β-glucosidase activity using *p*NPL and *p*NPG, respectively. The amount of protein produced by QM6a in this experiment was too low to include this cocktail in the comparison.

**Fig 4:**
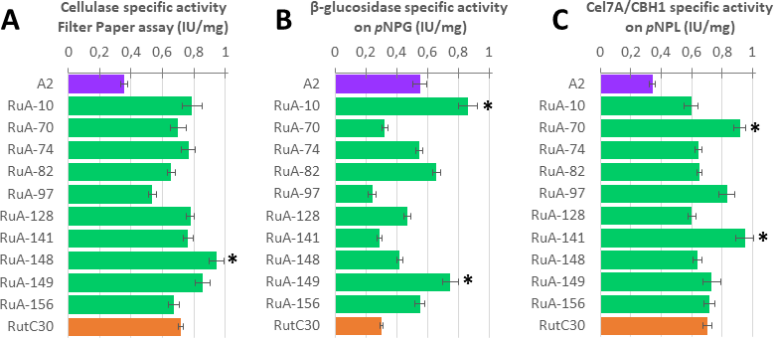
Specific enzymatic activities (IU/mg_Protein_) on A, filter paper (global cellulase) B, *p*NPG (β-glucosidase) C, and *p*NPL (Cel7A/CBH1). Error bars represent the standard deviation from the mean, for *n* = 3 technical replicates. Asterisks indicate significant differences compared with both parents A2 and RutC30 (Student’s *t*-test with *P*-value < 0.05).

The parental strains A2 and RutC30 displayed significantly different specific activities, with cellulase and cellobiohydrolase activities two times higher in RutC30, and conversely, β-glucosidase activity two times higher in A2 (Fig. 4). Most of the progeny strains showed specific activities in the range of their parents, but surprisingly, for such a small subset, we could identify at least one strain with a transgressive phenotype for each of the measured activities. Compared with the best parent, significantly higher activities are observed for RuA-148 (cellulase activity), RuA-10 and RuA-149 (β-glucosidase activity), and RuA-70 and RuA-141 (cellobiohydrolase activity) (asterisks in Fig. 4). Thus, genomic shuffling through sexual recombination allowed us to generate a new set of biodiverse strains, which is particularly important for β-glucosidase activity since its deficiency in the *T. reesei* cocktail is one of the bottlenecks of cellulose hydrolysis (58).

Interestingly, the extreme phenotypes observed in our experiments are not restricted to one strain but are distributed across five of the ten strains studied. This result suggests that it would be beneficial to design a broader phenotypic screen of all offspring to identify strains capable of producing enzyme cocktails with a wide range of activities.

### Three types of karyotypes are observed in the selected strains

To gain insight into the hyperproduction genotype of the 10 selected strains, their genomes were sequenced and mapped on the parental genomes. The RutC30 genome was constructed *in silico* from the QM6a assembly (27, 28, 47) using data from the literature to introduce mutations and structural rearrangements (30). Since the A2 sequence was not assembled into chromosomes, we preferred to reconstruct a new version *in silico* from the chromosome sequences of the CBS999.97 (1–1, *re*) ascospore (Genbank: Bioproject PRJNA352653), which were available at the time of this analysis. The translocation between chromosomes II and IV (47) was corrected to obtain the wild-type (QM6a-like) karyotype of A2. The polymorphism rate between A2 and CBS999.97 was then assessed by mapping the A2 sequences onto the reconstructed genome. Depending on the chromosome, the values ranged from 1.1E-05 to 4.2E-05 mutations/bp, confirming that this reconstructed genome is a relevant representative of A2. By contrast, the degree of variation between A2 and RutC30 is 1.6 per 100 nucleotides with an even distribution across the whole genomes.

**Fig 5:**
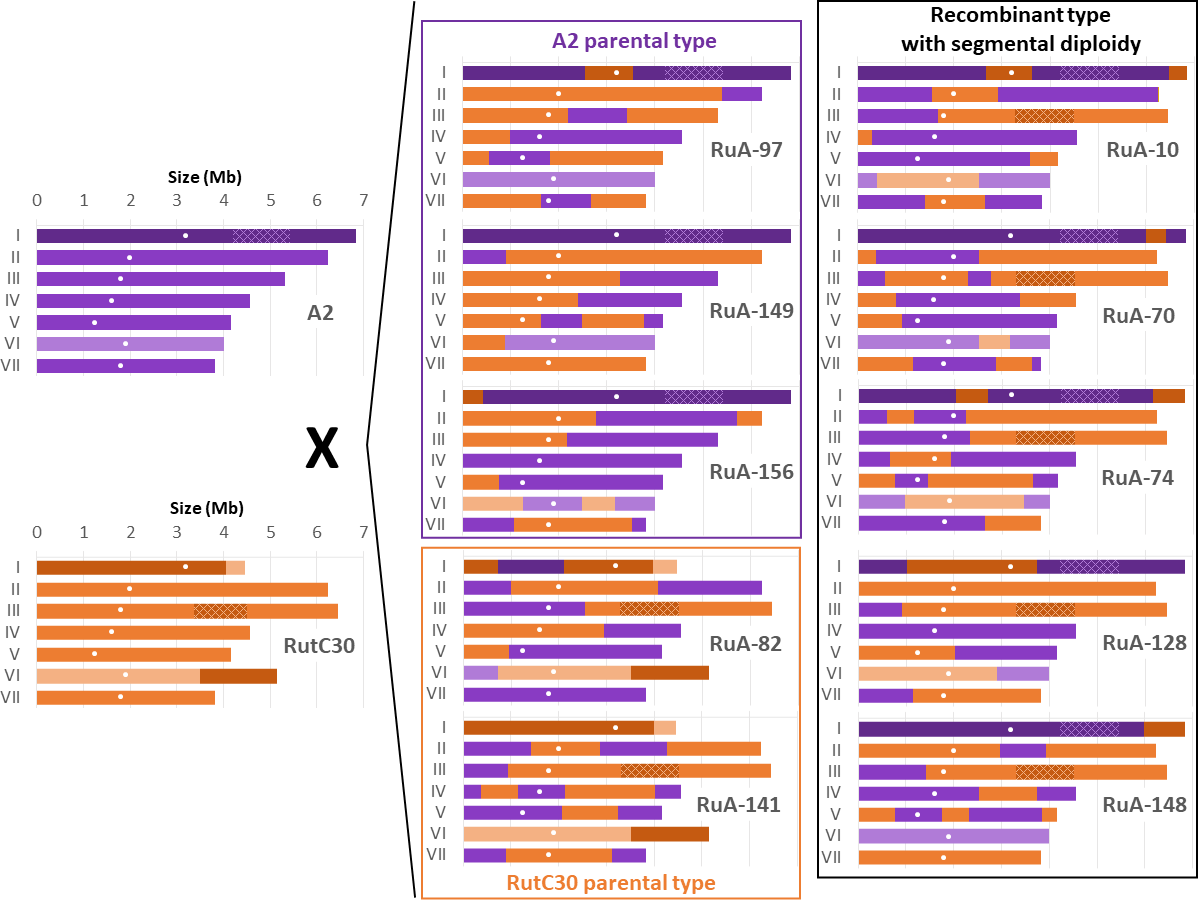
Karyotypes of the 10 selected hyperproducing strains reconstructed after mapping to parental genomes. A2 allelic fragments are in purple; RutC30 allelic fragments are in orange. To highlight the regions that translocated in RutC30, chromosome I fragments are in dark color, and chromosome VI fragments are in light color. The fragment of chr I translocated into chromosome III, resulting in segmental diploidy, is shown with hatching. Centromeres are shown as white dots

The progeny karyotypes were reconstructed by identifying the chromosomal regions of both parents according to the polymorphism rate (Fig. 5). As expected from (30), three patterns were identified: the parental types (A2 and RutC30), a recombinant type with an A2-like chromosome I, and a RutC30-like chromosome III. The strains with the latter karyotype showed segmental diploidy, with both allelic versions of the genes located in the translocated region from chromosome I to III in the RutC30 genome. None of the other translocated fragments were found to be duplicated or deleted in these descendants, which is consistent with the expected lethality of such karyotypes with segmental aneuploidy, as inferred by (30). Nevertheless, a broader population karyotype analysis would be required to be conclusive about the lethality of specific karyotypes. Segmental diploidy is found in half of the selected strains and involves 334 genes (TrA1450C to TrA1605C, File S1). The occurrence of second allelic versions could favor productivity by leading to overexpressed genes. In this segment, we noticed the gene *cel1a*, which encodes an intracellular β-glucosidase required for lactose induction and whose overexpression has been shown to increase the production of the cellobiohydrolase CBH1 (59). However, a transcriptomic study would be needed to get further information on the expression dosage of the genes present in the two allelic versions.

The number of crossing-over per chromosome ranges from 0 to 4, with most chromosomes having 1 or 2 crossing-overs (39% and 30% of the 70 sequenced chromosomes, respectively). Surprisingly, in RuA-10 and RuA-74, we observed two independent cases of simultaneous crossing-overs between two chromosome pairs. The RuA-10 karyotype results from two crossovers between both A2 and RutC30 chromosomes I, two crossovers between both RutC30 and A2 chromosome VI, and one crossover between A2 chromosome I and RutC30 chromosome VI. Similarly, the RuA-70 karyotype is caused by crossovers between RutC30 chromosome VI and both A2 chromosome I and VI. This unusual event is a consequence of the translocated region from chromosome I to chromosome IV in RutC30. No recombination event was observed in the right arm of RutC30 chr III, which could be due to the presence of both the translocated region and the NRR surrounding the *MAT* locus.

### Enrichment of specific RutC30 mutations in the higher producers

In an attempt to elucidate the sources of the hyperproductivity of the 10 selected strains, the allelic version of all genes was determined, and the chromosomal regions enriched in specific versions were identified (genes in at least 8 of 10 strains, File S1). When the enriched region is from the RutC30 parental strain, the mutated genes included in the area may be referred to as advantageous for cellulase production while the depleted regions could be referred to as deleterious. The diploid segment mentioned above has been excluded from this analysis. The results of this investigation are summarized in Table 3. Among the 17 regions with uneven allelic versions, 10 are depleted and seven are enriched in the RutC30 version.

**TABLE 3:** Genome-wide analysis of the allelic frequency in the genotype of the 10 selected strains: identification of regions enriched in either A2 or RutC30^*b*^

ID region	Chromosomal region	Allelic frequency of RutC30	Genes number	ID mutated genes	Mutated element	Function
I.1	TrA0149C - TrA0654C	10% and 20%	489	TrA0236W	Intron	Putative adaptor protein complex AP-1 medium subunit
				TrA0325W	Exon	Putative RNA small subunit methyltransferase
				TrA0345W	Promoter	Putative glycoside hydrolase family 31 GLS2
				TrA0564W	Promoter	Putative protein of unknown function
I.2	TrA1093W - TrA1247C	20%	134	TrA1148C	Exon	Putative MYB transcription factor
I.3	TrA1606W - TrA1804C	20%	178	TrA1627W	Promoter	Putative protein of unknown function
				TrA1660W	Exon	Putative glycerol-3-phosphatase GPP1
				TrA1723W	Intron	Putative lysine-specific histone demethylase
II.1	TrB0543W - TrB0931W	80%, 90% and 100%	365	TrB0564W	Promoter	Putative protein of unknown function
				**TrB0655C**	**Exon**	**Karyopherin KAP8**
				TrB0668W	Promoter	Putative aconitase hydratase
				**TrB0812W**	**Exon**	**Transcription factor ACE3**
				TrB0915C	Promoter	Putative protein of unknown function
III.1	TrC0505C - TrC0618W	80%	111	TrC0547W	Exon	Putative protein of unknown function
				TrC0575W	Promoter	Putative protein of unknown function
III.2	TrC0796C - TrC0933W	80%	127	TrC0796C	Exon	Putative protein of unknown function
				TrC0885C	Intron	Putative protein of unknown function
				**TrC0903C**	**Exon**	**Carbon catabolite repression transcription factor CRE1**
				**TrC0909W**	**Promoter**	**Transcription factor MH25**
				TrC0933W	Promoter	Putative Zn2Cys6 transcription factor
III.3^*a*^	TrC0985C - TrC1580C	80%	557	TrC1111W	Exon	Putative protein of unknown function
				TrC1117W	Exon	Putative MYB transcription factor
				TrC1143W	Terminator	Putative DNA lyase
				TrC1344W	Exon	Putative Zn2Cys6 transcription factor
				TrC1485W	Exon	Putative MFS permease
				TrC1488W	Intron	Putative protein of unknown function
				TrC1498W	Promoter	Putative peptide synthetase
IV.1	TrD0593C - TrD0657W	20%	59	TrD0606W	Promoter	Putative protein of unknown function
				TrD0618W	Promoter	Putative protein of unknown function
				TrD0646W	Exon	Putative oligomeric Golgi complex component
IV.2	TrD0938W - TrD1067W	20%	117	TrD1009C	Promoter	Putative MFS permease
				TrD1050W	Promoter	Putative HET domain protein
IV.3	TrD1186C - TrD1442C	10% and 20%	243	TrD1226W	Exon	Putative protein of unknown function
				TrD1323C	Terminator	Putative protein of unknown function
V.1	TrE0001C - TrE0159C	80%	147	**TrE0103W**	**Exon**	**Zn(2)Cys6 transcription factor involved in β-glucosidase expression BglR**
V.2	TrE0312W - TrE0426W	20%	104	none		
V.3	TrE0484C - TrE0518W	20%	32	TrE0504C	Exon	Putative B-type cyclin involved in cell cycle progression CLB4
V.4	TrE1164W - TrE1200W	20%	35	none		
VI.1	TrF1053W - TrF1210C	20%	149	TrF1147C	Exon	Putative ABC transporter
				TrF1184C	Promoter	Putative A/G-specific adenine glycosylase
VII.1	TrG0802C - TrG0831C	80%	29	none		
VII.2	TrG0864W - TrG0946W	80%	79	TrG0888W	Intron	Putative protein kinase

^*a*^ This region includes the non-recombination area.

^*b*^ A2-enriched regions are in blue, RutC30 in gray, and RutC30-enriched region in white. Genes in bold are directly related to cellulase production.

Interestingly, only a single and short region of chromosome II originating from the RutC30 is shared by all 10 strains. Among the 28 genes (TRB0802W to TrB0830W) located in this region, we identified one single-mutated gene encoding the transcription factor ACE3. As already mentioned, the RutC30 strain contains a truncated version of *ace3,* resulting in increased expression of several cellulolytic genes compared with the wild type (32). This chromosomal fragment is surrounded by a large zone with an allelic frequency of 90%, which itself is encompassed by a zone of 80%. Four other mutated genes were identified in the region, but only one in the exon element. This gene encoding the ß-importin KAP8 has been shown to be essential for the nuclear import of the main cellulase transcription factor XYR1 (60). Two other enriched regions can be directly linked to mutations advantageous for cellulase production: region III.2 with the truncated version of *cre1* and region V.I with an SNP in the gene encoding the transcription factor *BglR*. This last mutation has not been studied in the RutC30 genetic background, but a missense mutation of *BglR* in the PC3-7 strain results in increased cellulase production (34). In RutC30, the SNP leads to the replacement of a glutamate by a glycine at position 260, eliminating the prediction of the fungal-specific transcription factor domain (position 216–286, ID Smart: SM00906). Among the depleted regions, no mutated genes appear to be deleterious for production and thus counter-selected. However, we cannot exclude that the enriched A2 allelic version *per se* (i.e., without mutation) could be advantageous for hyperproductivity.

In an attempt to identify the sources of improvement in β-glucosidase and cellobiohydrolase activities, the allelic version of the genes encoding for these enzymes was determined. *T. reesei* has 11 putative β-glucosidases (61), but Cel3A/BGL1 is the most important player under induction conditions (62). The two strains with increased activity carried different alleles: the RutC30 version for RuA-10 and the A2 version for RuA-149, suggesting that there is no relationship between the allelic version of *bgl1* and the observed phenotype. Similarly, we cannot detect a specific pattern of the other 10 genes encoding β-glucosidases for RuA-10 and RuA-149 (Supplementary Data File 1). As the pNPL measurement is a portrayal of Cel7a/CBH1 performance, we determined the allelic version of *cbh1* in the two top-performing strains and found that RuA-70 has the RutC30 version, whereas RuA-141 has the A2 version. Once again, the allelic version is not a source of improvement. The phenotype found in these strains is therefore due to differences in the enzymatic cocktail composition and not in enzyme-specific activities. A transcriptomic study performed under production conditions would provide additional information to understand the basis of these phenotypes.

### Ability of the selected strains to sexually reproduce

As the ability to reproduce sexually is a promising way to improve industrial strains, it would be convenient to use these strains in new further rounds of improvement by crossing. The fertility of the selected strains was therefore tested by crossing them with the fertile female strains A2 and B31 and the sterile female strains QM6a *MAT1-1* and *MAT1-2* (Table 4). As expected, all strains produced ascospores when mated with a compatible fertile female strain. In contrast, five of 10 crosses with a compatible sterile female strain produced stromata, and only the two *MAT1-1* strains (RuA-149 and RuA-156) generated mature and fertile stromata capable of ejecting ascospores. Since the basis for the female sterility of QM6a and, consequently, RutC30 is a defective *idc1* (36), we investigated which allele is present in the progeny. The five strains in which stromata formation was observed carry a functional version of *idc1*. The introduction of a functional *idc1* gene in the QM6a strain is sufficient to restore fertility, but no complementation experiment of the mutated version of *idc1* with a functional one in RutC30 has been reported. Therefore, we might speculate that the infertility of the three *MAT1-2* strains (RuA-71, RuA-74, and RuA-148) is due to mutations affecting fertility already present in the RutC30 strain. For instance, one of the translocations (29) between chromosome I and III truncates the STE-like transcription factor gene *pp-1* (ID36543/TrA1391C). In *N. crassa*, deletion of the *pp-1* ortholog (NCU00340, 91% of identity with TrA1391C) results in the inability to form viable ascospores (63). The three sterile *MAT1-2* strains carry both the mutated and wild-type alleles while the fertile female strains have only the wild-type ones. A complementation experiment would be required to verify this hypothesis.

**TABLE 4:** Fertility tests on the 10 selected strains^*a*^

Strains	Mating type	X A2	X B31	X QM6a *MAT 1–1*	X QM6a *MAT 1–2*	*idc1* version
RuA-10	*MAT1-2*	F	S	S	S	*idc1nf*
RuA-70	*MAT1-2*	F	S	S	S (stromata)	*idc1f*
RuA-74	*MAT1-2*	F	S	S	S (stromata)	*idc1f*
RuA-82	*MAT1-2*	F	S	S	S	*idc1nf*
RuA-97	*MAT1-2*	F	S	S	S	*idc1nf*
RuA-128	*MAT1-2*	F	S	S	S	*idc1nf*
RuA-141	*MAT1-2*	F	S	S	S	*idc1nf*
RuA-148	*MAT1-2*	F	S	S	S (stromata)	*idc1f*
RuA-149	*MAT1-1*	S	F	S	F	*idc1f*
RuA-156	*MAT1-1*	S	F	S	F	*idc1f*

^*a*^ The fertility of the 10 strains was tested by crosses with female fertile strains (A2 or B31) and female sterile strains (QM6a *MAT1-2* or *MAT1-1*). Sterile crosses are indicated by an S and fertile crosses by an F. The functional version of *idc1* is noted as *idf1f* and non-functional as *idc1nf*.

The two fertile female strains display a different karyotype from the parental strain RutC30. It has already been shown that the crossing of strains with chromosomal alterations leads to non-viable segmental aneuploidy spores (21). Therefore, it would be of interest to identify fertile female *MAT1-1* strains with a RutC30-type karyotype in the progeny and thus have a valuable tool strain for future experiments. As the karyotypes of other industrial strains derived from QM6a (namely the QM9414 lineage) have also undergone translocations (30), a similar experiment could also be carried out.

Since RuA-149 is compatible with all the other industrial strains because of *MAT1-1*, we tested the possibility of crossing it with strains other than QM6a. Fertile crosses were obtained with strains NG14, RutC30, QM9414 (64), and Tu-6 (65) (data not shown).

### Conclusion

In this work, we conducted the first experiment of sexual outbreeding in *T. reesei* and demonstrated that this approach allows the generation of outperforming strains compared with the parents. The approach described in this article requires both high-throughput screening to test large numbers of individuals and efficient selection to differentiate between individual phenotypes. These constraints are of the same order as those encountered when optimizing strains by random mutagenesis. Nevertheless, a further advantage associated with sexual reproduction via outbreeding over random mutagenesis, which results in the accumulation of deleterious mutations and genetic engineering necessitating the recycling of the selective marker, is the ability to implement an iterative optimization process without any limit on the number of cycles. The combination of these three approaches could be employed to enhance the efficacy of the improvement process, thereby mitigating the limitations of each approach in isolation.

## SUPPLEMENTAL MATERIAL

Supplemental figures spectrum.00441-24-s0001.pdf Fig. S1-S3.

Supplementary File S1 spectrum.00441-24-s0002.xlsx Allelic version of the genes in the 10 selected strains.

ASM does not own the copyrights to Supplemental Material that may be linked to, or accessed through, an article. The authors have granted ASM a non-exclusive, world-wide license to publish the Supplemental Material files. Please contact the corresponding author directly for reuse.
