# Supplementary material for: Transgressive phenotypes from outbreeding between the Trichoderma reesei hyper producer RutC30 and a natural isolate
Source: Microbiol Spectr. 2024 Aug 20;12(10):e00441-24. doi: 10.1128/spectrum.00441-24 (PMC11448445; doi:10.1128/spectrum.00441-24)
Supplement: Supplemental figures — Fig. S1-S3. [file spectrum.00441-24-s0001.pdf]

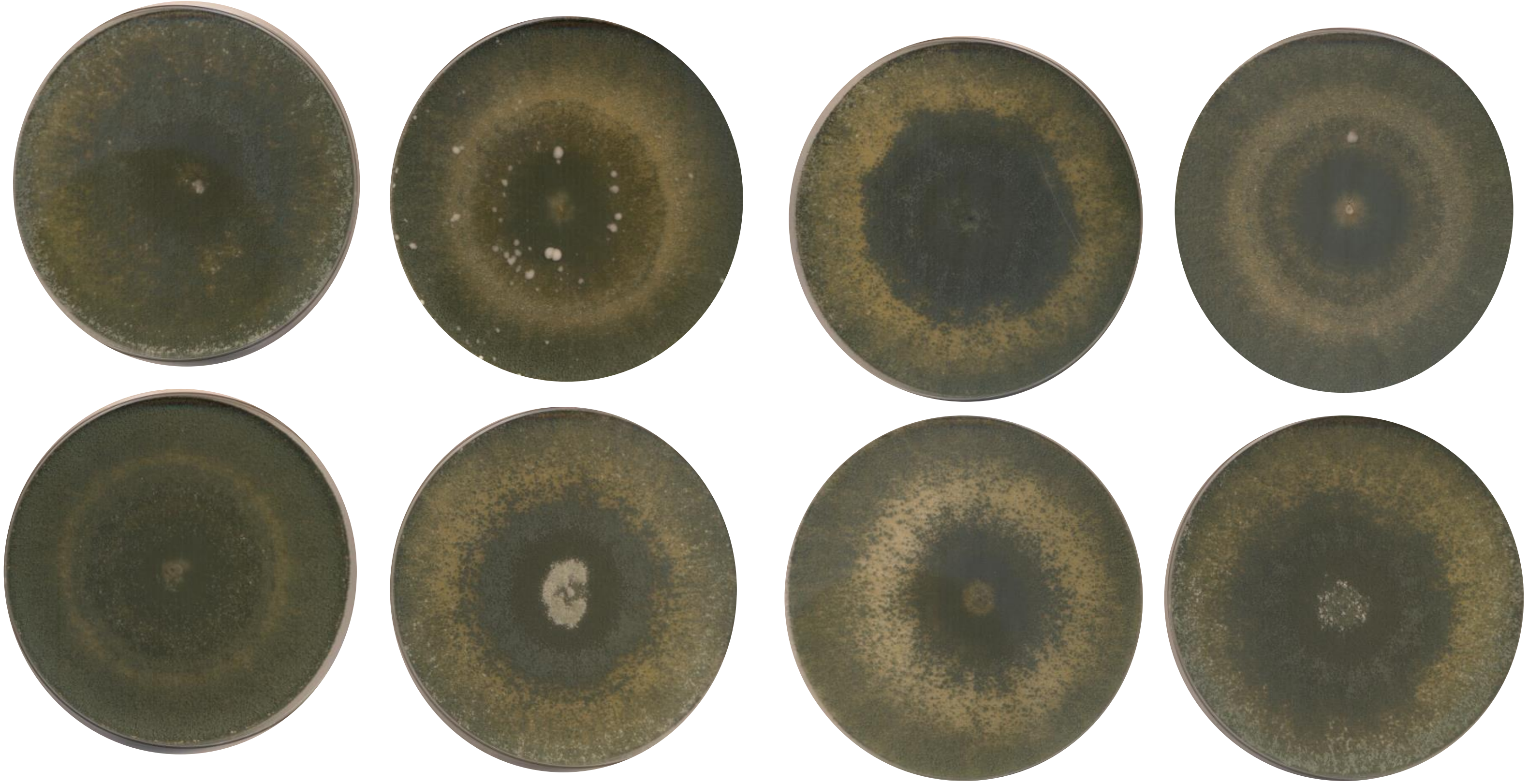

CBS999.97

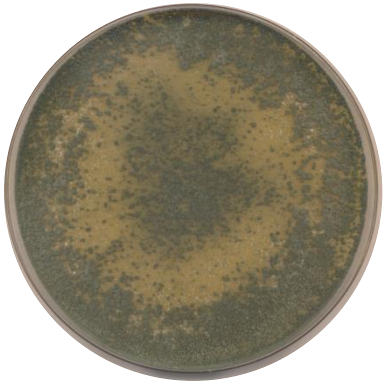

Figure S1: Phenotypes of the progeny from CBS999.97. Ascospore-derived isolate and the parental strain were grown cultivated on PDA for 7 days at 30°C with an alternance of 18 hours of light for 6 hours of darkness

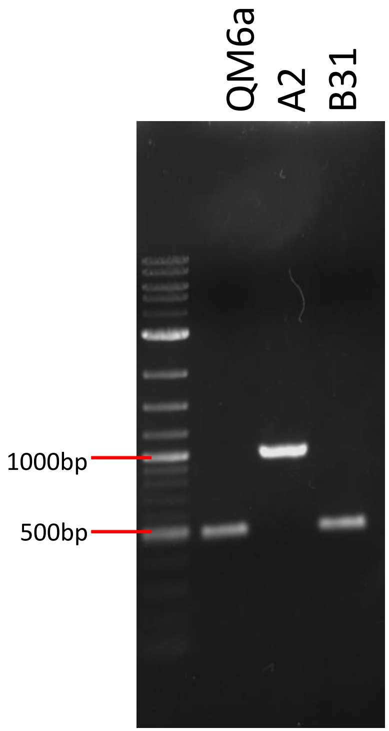

Figure S2: Duplex amplification of an internal fragment of the *MAT1-1* (995 bp) and *MAT1-2* (500 bp) loci. Line 1: QM6a, line 2: A2, line 3: B31. The oligonucleotides used for amplification are summarised in Table S2

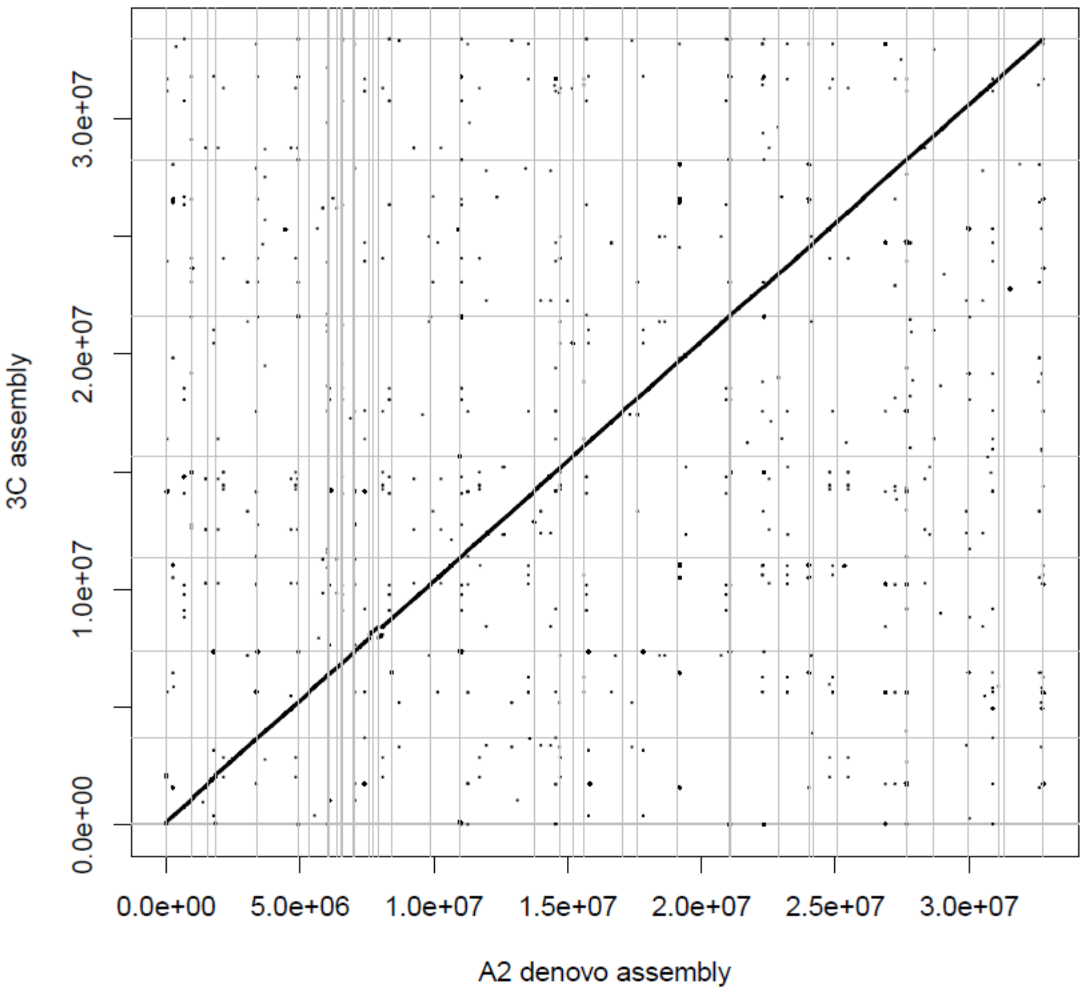

Figure S3: Similarity matrix between A2 and QM6a strains

Comparison of contigs from the *de novo* assembly of strain A2 and the reassembled sequence of strain QM6a (30). Contigs and chromosomes resulting from the assembly of the A2 and QM6a were concatenated and compared. The horizontal lines correspond to the chromosome ends of the QM6a assembly and the vertical lines correspond to the contigs of A2 assembly. The contigs of the A2 strain were ordered and inverted where necessary to obtain a diagonal.
